# Numerical Modeling and Analysis of Ti6Al4V Alloy Chip for Biomedical Applications

**DOI:** 10.3390/ma13225236

**Published:** 2020-11-19

**Authors:** Waqas Saleem, Bashir Salah, Xavier Velay, Rafiq Ahmad, Razaullah Khan, Catalin I. Pruncu

**Affiliations:** 1 Department of Mechanical and Manufacturing Engineering, Institute of Technology, F91 YW50 Sligo, Ireland; velay.xavier@itsligo.ie; 2 Industrial Engineering Department, College of Engineering, King Saud University, P.O. Box 800, Riyadh 11421, Saudi Arabia; bsalah@ksu.edu.sa; 3 Department of Mechanical Engineering, University of Alberta, Edmonton, AB T6G 1H9, Canada; rafiq.ahmad@ualberta.ca; 4 Department of Mechanical Engineering Technology, University of Technology, Nowshera 24100, Pakistan; razaullah@uotnowshera.edu.pk; 5 Manufacturing & Engineering Management, University of Strathclyde, Glasgow G1 1XJ, UK; 6 Faculty of Engineering, Department of Mechanical Engineering, Imperial College London, London SW72AZ, UK

**Keywords:** Ti6Al4V, Johnson-Cook, simulation of cutting processes, chip morphology

## Abstract

The influence of cutting forces during the machining of titanium alloys has attained prime attention in selecting the optimal cutting conditions to improve the surface integrity of medical implants and biomedical devices. So far, it has not been easy to explain the chip morphology of Ti6Al4V and the thermo-mechanical interactions involved during the cutting process. This paper investigates the chip configuration of the Ti6Al4V alloy under dry milling conditions at a macro and micro scale by employing the Johnson-Cook material damage model. 2D modeling, numerical milling simulations, and post-processing were conducted using the Abaqus/Explicit commercial software. The uncut chip geometry was modeled with variable thicknesses to accomplish the macro to micro-scale cutting by adapting a trochoidal path. Numerical results, predicted for the cutting reaction forces and shearing zone temperatures, were found in close approximation to experimental ones with minor deviations. Further analyses evaluated the influence of cutting speeds and contact friction coefficients over the chip flow stress, equivalent plastic strain, and chip morphology. The methodology developed can be implemented in resolving the industrial problems in the biomedical sector for predicting the chip morphology of the Ti6Al4V alloy, fracture mechanisms of hard-to-cut materials, and the effects of different cutting parameters on workpiece integrity.

## 1. Introduction

Ti and its alloys exhibit excellent material characteristics and are widely used in aerospace, marine, and medical applications. Their distinguishing characteristics include substantial strength-to-weight ratio, low thermal conductivity, excellent fatigue performance [1], exceptional corrosion resistance [2], low elastic modulus, high yield strength up to 550–600 °C, good cryogenic properties [3], and excellent biocompatibility compared with stainless steel [4]. In the biomedical sector, these characteristics make them an ideal candidate for the fabrication of orthopedic, cardiovascular and dental implants, as well as surgical instruments, jigs and fixtures [5]. Due to the high chemical affinity for oxygen, a corrosive resistant layer of TiO_2_, with some other oxidation states such as Ti_2_O_3_ and TiO, is produced on the machined Ti implants [6]. The thickness of this thin passive layer ranges from 3 to 6 nm, and transforms the titanium alloys into biologically inert metals that can be implanted into the body for more than twenty years. Moreover, the non-ferromagnetic property of Ti alloys makes them safe for examinations with technologies such as magnetic resonance imaging (MRI) [7].

Besides the excellent material characteristics, titanium, and its alloys are considered difficult-to-machine materials with poor machinability [8]. Machining of Ti-based biocompatible materials for implants imposes challenges because of the difficulties in attaining intricate geometrical profiles with exceptional surface quality. The quality of machined implants influences its long-term application. For example, the surface roughness and module of elasticity of Ti alloys orthopedic implants directly manipulates the bone response. It is possible to promote osseointegration with an enhanced surface roughness [9]. Because of the low conductivity of Ti alloys, most of the heat produced during machining operations is transferred to the tool cutting edge [10]. Therefore, machining is performed with carbide cutting edges having specialized coatings such as TiAlSiN or TiAlN to achieve the desired surface finish [11]. However, prolonged exposure of the cutting edge to the elevated temperature damages the hard-wearing layer and advances rapid chemical reactions which limit the material removal rate. Machining induced residual stresses also influence fatigue and corrosion resistance of the implants.

The cutting tool wear is a vital problem under dry cutting conditions due to increased thermal loads and the development of machining induced tensile residual stresses which increase surface damage [12]. Due to their low elastic modulus, titanium alloys also demonstrate a spring-back action during machining, and tend towards poor surface quality due to chattering [13]. Since tool wear is a prominent problem in the machining of Ti6Al4V, numerous numerical and experimental studies have been undertaken to understand the chip morphology in order to enhance the tool life and workpiece surface integrity. Venugopal et al. [14] considered the effect of tool wear during machining of grade 5 Ti alloys. Their study showed that the formation of wear craters occurred due to the adhesion and diffusion mechanisms. Up until now, in numerical studies, the mechanics of the Ti-6Al-4V chip have not been well understood, as they show drastic changes in chip formation if parameters are changed rationally. The serrated chip morphology in Ti6Al4V cutting is influenced by adiabatic shearing [15]. The serration effect becomes more prominent with high cutting speeds [16]. Substantial research efforts have been made to establish the optimized cutting parameters of Ti6Al4V by means of laser [17], cryogenic [18], electric discharge machining (EDM) [19], and advanced milling [20]. From different studies, it has been observed that surface integrity and dimensional accuracy are influenced by milling speed. Optimal results are achieved if the cutting speed is kept below 100 m/min [21].

Ti6Al4V’s strength is reduced considerably if the temperature rises above 800 degrees Celsius. This effect is more prominent in the case of dry machining where temperatures rise above 900 degrees Celsius [14]. This effect subsequently results in increasing thermal softening and hence the strain hardening. Due to the increased strain hardening, the strain tries to confine in a narrow band, normally called a shear band [16]. Such a high temperature at the tool-chip interface demands the use of an appropriate coolant and lubrication during the cutting action. Hoyne et al. [22] investigated the effects of an atomization-based cutting fluid on cutting temperatures with Ti alloys. They demonstrated that the spray system was more effective in reducing cutting temperatures than flood cooling or dry conditions.

Ramana et al. [23] studied the chip morphology of grade-5 Ti alloys with different process parameters under dry, flooded, and minimum quantity lubrication (MQL) conditions. It was detected that MQL machining produces promising results by reducing the temperature at the tool-chip interface. Ye et al. [24] investigated the evolution of chip configuration. It was reported that the conversion of a continuous chip to a serrated one is accomplished by the formation of repetitive shear bands. Sun and Guo [25] investigated the milling parameters of Ti alloys and evaluated that the serrated tooth chips and saw-tooth frequency are inversely related, and that this greatly depends on the cutting velocity. Zhang et al. [26] concluded that the cutting velocity has a more prominent effect on the rising of the temperature of the serrated chip as compared to the rake angle. It is expected that the recent developments in cryogenic cooling will resolve the elevated temperature problems during the machining of Ti alloys.

Studies based on numerical cutting simulations have evaluated the likely mechanisms involved in the formation of segmented chips of Ti alloys. In the published studies, cutting forces predicted through milling simulations have shown good agreement with the experimental data. However, the accuracy of a numerical cutting simulation relies on the quality of the inputs and models, such as: reliable thermo-mechanical material properties, the material constitutive model, the contact friction model, the strain and strain rate history, the viscosity and thermal loading history, and the material damage criteria. The relevant constitutive models include Oxley’s constitutive model [27], the Power-law model [28], the strain path-dependent model [29], and the Johnson-Cook (JC) model [30]. Among these, the Johnson-Cook constitutive material model is most vital as it integrates different formulations, flow stress, strain, strain-rate, and temperature. The JC material damage model has demonstrated reliable results in nonlinear modeling of various cutting operations.

Researchers have addressed comprehensive numerical studies on the thermo-mechanical phenomena involved at the tool-chip interface during Ti-6Al4V machining [31]. The numerical chip formation in the Ti6Al4V alloy is supported by three established theories. According to the first theory, chip evolution takes place through the adiabatic shear band instigated by the thermal and strain softening effect in the material [32]. The second theory considers crack propagation along the shear plane [33] initiated at the tool-tip. The third theory considers the effects of both actions, in which the adiabatic shear band directs the crack propagation [34]. For Ti6Al4V, researchers have contributed meticulous efforts to determine the Johnson-Cook damage parameters to be employed realistically in numerical models. One of the methodologies uses compression tests by a split-Hopkinson pressure bar [35]. The reported parameters show considerable difference in values, and hence a substantial variation is observed in numerical outcomes when a particular set of parameters is employed on the same finite element model. Ducobu et al. [36] presented a comprehensive research study by undertaking twenty collections of Johnson–Cook parameters reported in the literature for the Ti6Al4V alloy. The work was focused on identifying the most illustrative set of parameters that best corroborated the outcomes of the numerical cutting model with the experimental results.

One of the issues involved with the cutting simulation of Ti6Al4V is the formation of segmented chips at high cutting speeds. Jiang et al. [37] described that this particular phenomenon in Ti6Al4V chip formation occurs because of the plastic instability in the primary shear zone due to thermal softening and the work hardening effect. Mehmet et al. [38] predicted flat-end milling forces by analyzing the chip morphology and cutting force in the high-speed cutting of Ti6Al4V alloy. Chen et al. [39] worked on the JC failure model with an energy-based failure criterion for high-speed cutting of Ti6Al4V for different cutting conditions. Nithyaraaj et al. [40] studied the modeling and simulation of the orthogonal cutting process by using different sets of constitutive model parameters and investigating their influence on the outcome. The authors analyzed the reaction forces and chip morphology and validated the results with experimental orthogonal cutting references. Hou et al. [41] proposed a modified Johnson-Cook model by introducing a temperature function into the work hardening term. The main objective was to incorporate a temperature-dependent hardening effect for smooth flow stress behavior of the Ti6Al4V alloy under a set of dynamic loading conditions.

In aerospace and medical applications, the milling of Ti alloys supersedes other compatible machining operations to attain productivity. Numerical modeling with finite element analysis of milling at macro and micro-scale is challenging, and demands extensive parametric sensitivity analyses to establish the optimized parameters for a complex thermo-mechanical interaction at the tool-chip interface. In micro milling studies, chip morphology is greatly characterized by the cutting size effect, and leads towards a nonlinear increase in the specific cutting energy when the uncut chip thickness approaches a few microns [42]. Under this condition, a smaller shear angle dissipates more energy due to plastic deformations. To accomplish the micro-milling cutting action, the cutting feed must approach a minimum threshold level to initiate a crack and chip off the material [43].

Besides the finite element cutting simulations, researchers have explored other predictive modeling techniques to understand Ti6Al4V chip morphology, cutting reaction forces, and thermal effects. These include hybrid modeling approaches, molecular dynamics, and multi-scale modeling [44,45]. In the case of finite element (FE) milling simulations, predictive modeling techniques become quite challenging due to certain factors. In a milling simulation study, it is imperative to consider the effect of tool edge radius, the trochoidal trajectory of the cutting flute, and the tool-spindle run out [46]. Saleem et al. [47] worked on the macro and micro-scale numerical modeling by analyzing the effects of various cutting parameters (cutting speed, feed rate, tool geometry, etc.) in order to consistently control the tool-chip interaction. Li et al. [48] developed an iterative algorithm to evaluate the chip thickness by integrating supplementary factors in the constitutive model, as well as the cutting flute trochoidal trajectory, minimum chip thickness, and tool run-out. Likewise, Newby et al. [49] investigated the kinematics of chip formation by considering a trochoidal tool flute trajectory. Lai et al. [50] investigated the cutting reaction force in the milling process by employing the size effect. Kai et al. [51] presented temperature and stress distribution plots of a tungsten-carbide micro-milling tool. Karla et al. [52] performed a detailed study on milling characteristics of titanium, and stainless steel.

In addition to the numerical cutting simulations, researchers also applied statistical and heuristic optimization methods to determine the optimum milling parameters for difficult-to-cut materials at macro and micro scales. For example, Danish et al. (2020) [53] used response surface methodology (RSM) to predict the surface roughness of the Ti6Al4V alloy. Likewise, Lu et al. (2018) [54] applied RSM to evaluate the milling induced surface roughness. Shahriar et al. (2017) [55] implemented optimization schemes to determine the optimum milling parameters and simulated annealing, using the artificial bee colony and ant colony optimization algorithms. Saleem et al. (2017) [56] developed a numerical model in Abaqus and performed a parametric sensitivity analysis. The optimum cutting parameters were obtained by means of an Artificial Neural Network scheme.

Normally, for a cutting simulation, a 2D numerical study is preferred due to computational limitations and convergence of results. The chip formation process is supported by means of a coupled thermo-mechanical analysis and material damage constitutive laws in some commercial finite element analysis (FEA) solvers, like finite-element computer code ABAQUS. Chip segmentation by shear localization in machining of titanium alloys occurs due to thermo-plastic instability and ductile fracture. Element characteristics, mesh density, model boundary conditions, contact friction law, fracture parameters, features used in the numerical cutting model (such as tie constraint, coupling, self-contact conditions, mating contact conditions, etc.) and other assumptions adopted to converge the solution decide the initiation of material crack (damage of finite elements) and its propagation. The chip morphology follows the constitutive and damage parameters defined in the damage model. The selection of appropriate damage parameters plays an influential role to accommodate the high material strain and smooth stress flow. The modeling technique also affects the chip morphology. 

The main objectives of this research focus on the analysis of chip-morphology of Ti6Al4V alloy which is still not well understood. A comprehensive study of chip morphology is essential to select the optimum machining parameters to attain an excellent surface finish. In industrial applications, especially in the case of biomedical implants, subsequent processes like spray coatings, polishing, etc. are implanted to achieve the desired surface roughness (Ra) parameter. Likewise, stereo topography is also carried out to evaluate the microstructure of the machined implant surface. The numerical model developed for this simulation study considers the configuration of macro-to-micro scale milling chips, by using the Johnson-Cook constitutive damage model in Abaqus/Explicit. This research work is presented in form of a comprehensive numerical analysis to undertake the effect of chip stress flow, equivalent strain, cutting reaction force, cutting zone temperature, contact friction, and chip morphology at the macro-to-micro scale level. The numerical simulations are performed with three difficult cutting speeds with the same geometry of the cutting tool which does not undertake the tool wear effect and hence limit the scope of this research. The optimal cutting conditions predicted through the comprehensive modeling and simulation methodology were found to be in close approximation with the experimental data. The main parameters focused in this study include the temperature at the tool-chip interface, cutting reaction force, the equivalent strain, and cutting speed. 

**Figure 1:**
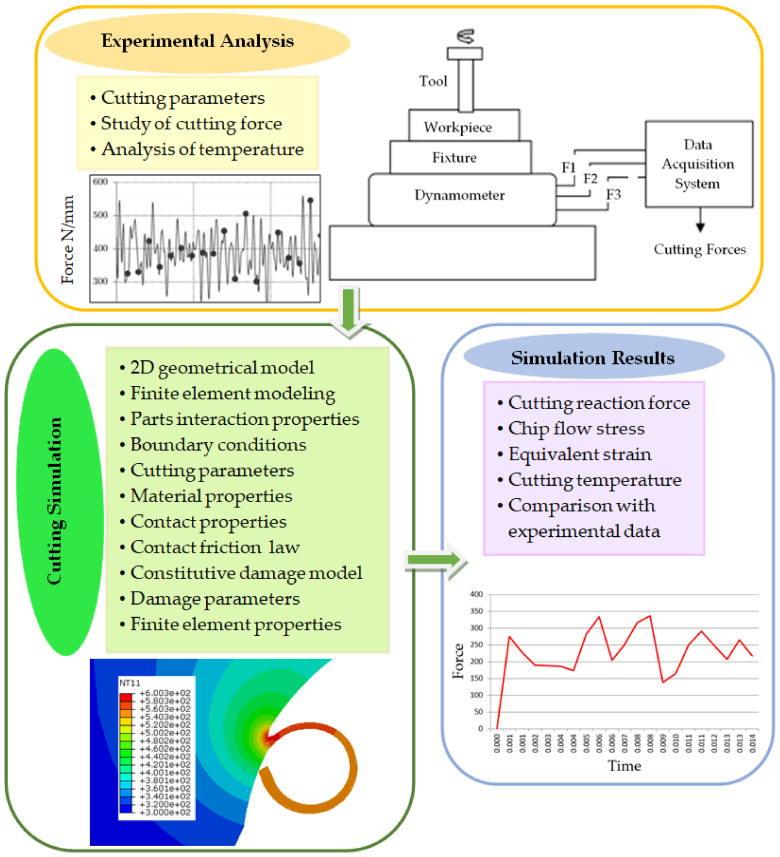
Graphical abstract of cutting simulation analysis.

This article is organized into five sections. In Section 1, a comprehensive literature review is presented which highlights the importance of Ti6Al4Va alloy for biomedical applications, different constitutive laws used in numerical cutting modeling, limitations of the existing numerical studies, and other important parameters related to the precision of numerical simulation results in case of difficult-to-cut materials, and novel aspects of the presented research study. Section 2 describes the numerical modeling and mathematical framework adopted for precise milling operation at macro-to-micro scale. Section 3 explains the methodology adopted, as well as analysis of the cutting simulations, discussion and conformance of results with the published data, and some novel aspects of the research. The important conclusions are given in Section 4. A graphical abstract of the cutting simulation analysis is given in Figure 1.

## 2. Numerical Approach

**Figure 2:**
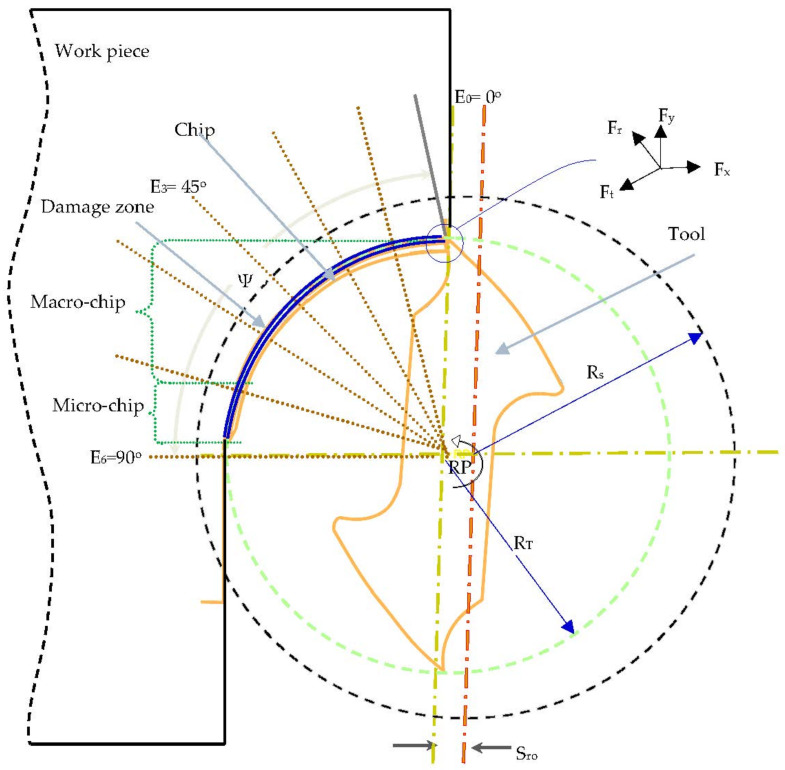
Schematic of cutting model.

The schematic of the cutting model used for the precise milling of Ti6Al4V is shown in Figure 2. The model takes on the uncut chip thickness by assuming a trochoidal trajectory of the milling cutter. The trochoidal trajectory directs the chip to follow a variable thickness as a function of angular position and feed [57,58]. The nomenclature used to explain the cutting configuration is as follows; ψ is the phase angle, O_t_ is the milling cutter center, R_T_ is the nominal radius of the milling cutter, S_ro_ is the spindle-tool run-out, and ω_r_ is the tool angular velocity (rad/s). The cutting tool experiences normal (F_r_) and tangential (F_t_) cutting forces. Forces along F_z_ are normally ignored due to their small contribution. If h is the thickness of the uncut chip at a specific time (t), then the minimum thickness at the primary shear zone (L_p_) is expressed as:

(1) $$h(t)=L_{p}\sin\phi$$

where, ϕ is the primary shear angle and is calculated as follows:

(2) $$\phi =\frac{\pi }{4}+\left[\frac{\gamma -\tan^{-1}\mu }{2}\right]$$

where, μ is the friction coefficient at the tool chip interface, and γ is the tool rake.

Milling simulations of the Ti6Al4V alloy were performed with the help of the Johnson-Cook constitutive damage model, which calculates the material flow stress by integrating the elasto-plastic behavior with strain hardening and thermal softening models [30]. The constitutive relationship is expressed as:

(3) $$\bar{\sigma }=(A+B{\bar{\varepsilon }}^{n})\left[1+C\ln\left(\frac{\dot{\bar{\varepsilon }}}{\dot{\bar{\varepsilon_{o}}}}\right)\right]\left[1-{\left(\frac{T-T_{room}}{T_{melt}-T_{room}}\right)}^{m}\right]$$

where, $\bar{\sigma }$ is the calculated stress, A is the yield stress, B is the hardening modulus, n is the work hardening coefficient, C is a constant for the strain hardening rate, m is the thermal softening coefficient, T_room_, and T_melt_ are the initial and melting temperatures of the material, $\bar{\varepsilon }$, $\dot{\bar{\varepsilon }}$ and ${\dot{\bar{\varepsilon }}}_{o}$ are the equivalent plastic strain, plastic strain rate and reference strain rate, respectively. 

The JC failure model contains five failure parameters D_1_, D_2_, D_3_, D_4_, and D_5_ which stand for the initial failure strain, exponential factor, triaxiality factor, strain rate factor and the temperature factor, respectively [30]. Damage is expressed by the following relation:

(4) $${\bar{\varepsilon }}_{oi}=\left[D_{1}+D_{2}\exp\left(D_{3}\frac{p}{\bar{\sigma }}\right)\right]\left[1+D_{4}\ln\left(\frac{\dot{\bar{\varepsilon }}}{\dot{\bar{\varepsilon_{o}}}}\right)\right]\left[1+D_{5}{\left(\frac{T-T_{room}}{T_{melt}-T_{room}}\right)}^{}\right]$$

Values of D_1_ to D_5_ for any specific material are determined experimentally and greatly depend on the testing method applied (Daoud, 2015) [59]. P is the average pressure stress. Hillerborg’s fracture energy-based criterion (G_f_) is implemented to initiate and propagate the material fracture, which can be expressed in terms of the fracture energy per unit area of the crack [60]:

(5) $$G_{f}=\int_{{\bar{\varepsilon }}_{oi}}^{{\bar{\varepsilon }}_{f}}L{\bar{\sigma }}_{y}d\bar{\varepsilon }=\int_{0}^{{\bar{u}}_{f}}{\bar{\sigma }}_{y}d\bar{u}={\bar{\sigma }}_{y}{\bar{u}}_{f}={\bar{\sigma }}_{y}\frac{2G_{f}}{\sigma_{f}}$$

where, L and $\overset{}{{\bar{u}}_{f}}$ are the characteristic length of an element and the equivalent plastic displacement, respectively (Baker, 2005) [61]. The fracture energy (G_f_) is expressed as a function of fracture toughness (K_C_) (Viggo, 2010) [62]:

(6) $${\left(G_{f}\right)}_{{}_{I,\;II}}=\left(\frac{1-\upsilon^{2}}{E}\right){\left(K_{C}^{2}\right)}_{I,\;II}$$

where, E is the modulus of elasticity, *v* is the Poisson ratio, K_IC_ and K_IIC_ are the mode I and II fracture toughness, respectively. For a scalar damage variable (D), the linear and exponential damage evaluation laws are given by [63,64]:

(7) $$\mathrm{Linear},\;D=\frac{L\bar{\varepsilon }}{{\bar{u}}_{f}}=\frac{\bar{u}}{{\bar{u}}_{f}}$$

(8) $$\mathrm{Exponential},\;D=1-\exp\left(-\int_{0}^{\bar{u}}\frac{\bar{\sigma }}{G_{f}}d\bar{u}\right)$$

In order to evaluate the effectiveness of the thermal aspects of the machining process, steady-state conditions should be achieved in the numerical simulation. Thermo-mechanical material properties and JC failure model parameters used in this research for Ti6Al4V are described in Table 1 and Table 2, respectively.

## 3. Numerical Cutting Simulation

**Figure 3:**
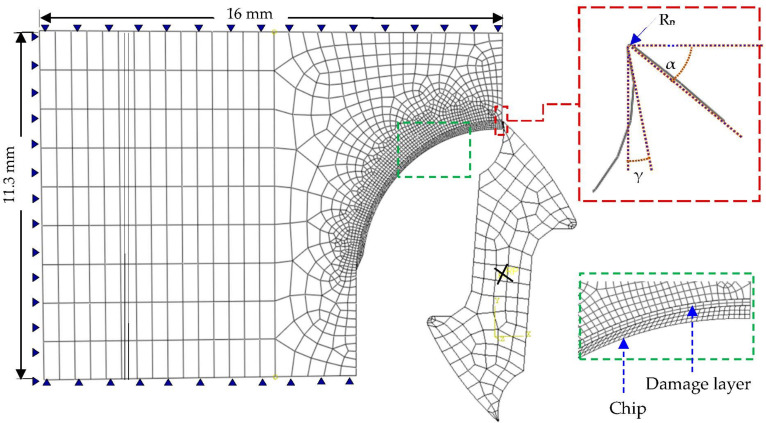
Mesh discretization of the tooling and workpiece.

The2D plane strain model with coupled thermo-mechanical analysis was used to simulate the macro-to-micro scale milling of the Ti6Al4V alloy by a commercial FEA solver ABAQUS. The finite element model and necessary boundary conditions are shown in Figure 3. The cutting insert was considered as a rigid material. The model is meshed with the element type PE4RT, a four-node quadrilateral continuum element with plane strain assumption, and coupled temperature-displacement. Appropriate mesh refinement is applied at the tooltip nose, material damage zone and the chip, in order to accommodate the high material strain and smooth stress flow. The thickness of the damage layer plays an influential role in deciding on the analysis step incremental time, and chip morphology, and it must be in accordance with the tooltip nose [68]. 

Adequate mesh density is necessary in order to achieve a compromise between computation time and strain localization. A refined mesh results in a smaller step time while a fine mesh density can lead to strain localization. In the presented study, a mesh size of about 20 to 30 µm is adopted at the contact interfaces. The chip geometry is modeled in such a way that the configuration of the uncut chip follows a macro scale thickness about three fourth sections during the cutting operation, and micro-scale at the end of one fourth of the cutting process. The cutting operation can be assumed as an orthogonal machining process, since the feed per tooth is considerably small compared to the tool diameter.

By employing a simple Coulomb’s friction law, two contact friction values (0.2 and 0.3) [16] were used at the cutting interfaces to comply with the relevant cutting speed. The analysis was used to predict the influence of cutting parameters on cutting reaction force, cutting zone temperature, chip stress, and the equivalent strain. The main features used in the numerical cutting model include: tie constraint (to fully integrate the workpiece, damaged layer, and the uncut chip), coupling (to integrate the cutting tool nodes with the reference point of rotation, RP), self-contact condition for the chip, tool contact condition with the chip, damage layer and the workpiece, and material initial conditions. The contact conditions were defined with the kinematic contact algorithm, by taking the tool as the master surface and chip front and lower sides as the slave surfaces. The geometry of the Tungsten carbide milling cutter is shown in Figure 3, in which γ is the rake angle (15°), α is the tool clearance angle (12°), and R_n_ is the radius at the tooltip nose (20 μm). RP is the tool reference point (RP) or the axis of rotation. The boundary conditions (angular velocity, the coupling of tool nodes, and fixed boundary conditions of the cutting tool) are defined with the reference point. From the numerical studies, it was evaluated that the model achieves the adiabatic steady-state conditions of about 0.2 ms. Three cutting simulation analyses were performed with different cutting speeds (21, 30 and 50 m/min), and two different contact friction coefficients (0.2 and 0.3). The predicted cutting reaction force, the temperature at the cutting zone, chip stress, and equivalent plastic strain were compared with the published data and experimental studies [16,40].

The numerical studies were verified by comparing with the experimental work performed by Ducobu et al. [69] on the orthogonal cutting of Ti6Al4V, under the same cutting conditions adopted in the model. The numerical studies were found to be in close approximation to the experimental results. A summary of the simulation results is presented in Table 3. A discussion and analysis of the cutting reaction force, the tool-chip interface temperature, the distribution of equivalent plastic strain (PEEQ), the chip morphology, and the material induced stresses are outlined below.

### 3.1. Discussion and Analysis

#### 3.1.1. Cutting Reaction Force

**Figure 4:**
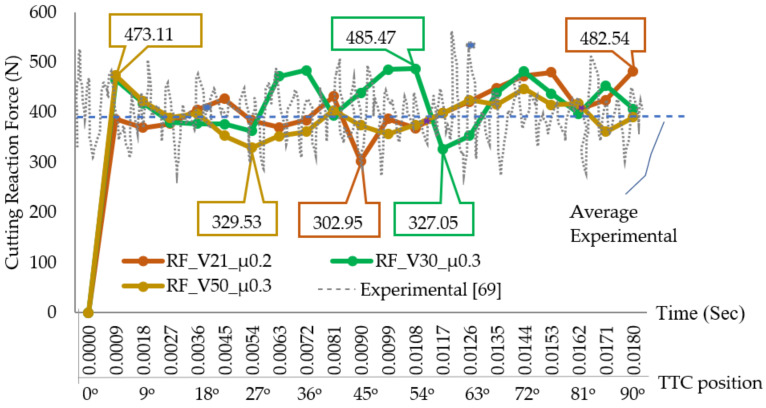
Simulated cutting reaction forces.

Figure 4 displays the simulated cutting reaction forces (RF), versus time for various cutting speeds (V) and contact friction coefficients (µ). For investigating the performance of the adopted predictive approach, the simulated forces are compared with the experimental forces. For similar cutting tool geometry and cutting parameters, details of the experimental setup for measuring cutting forces is given in the literature [69,70,71]. Cutting forces with respect to time were measured at the sampling frequency of 70 kHz using a Kistler 9257B dynamometer. The variations of the tangential cutting forces (F_t_) with cutting time occurred because of the periodic formation of the shear band. The average simulated cutting force with a cutting speed of 30 m/min was estimated to be 398 N, which successfully corroborates with the experimental results [69]. It was found that the contact friction coefficient has a great influence on heat generation and cutting reaction force. The average cutting reaction forces recorded with three different cutting speeds are presented in Table 4.

The absolute deviation between the simulated (387.96 N) cutting force at 30 m/min and measured average force (385 N) [69] at the same speed is estimated at 2.96 N, which shows the reliability of the numerical model. Overall, the simulated cutting force plots show a good accordance with the average experimental forces. Figure 4 also shows that the average cutting reaction forces remain in the specified bands for the various angular positions of the tooltip center (TCC). For example, at a cutting speed of 21 m/min, the cutting force ranges between 370 to 480 N. When the speed increases to 30 m/min, the cutting force is observed to be within a range of 360 to 408 N, while for 50 m/min the cutting force band is observed between 330 to 445 N. Considering this trend, it can be assumed that the cutting reaction force for Ti6Al4V falls between 350 to 450 N, depending upon the accuracy of the testing equipment, data acquisition systems, and material heat treatment process before the machining operations. The cutting tool experiences more reaction forces at lower cutting speeds.

#### 3.1.2. Chip Flow Stress 

**Figure 5:**
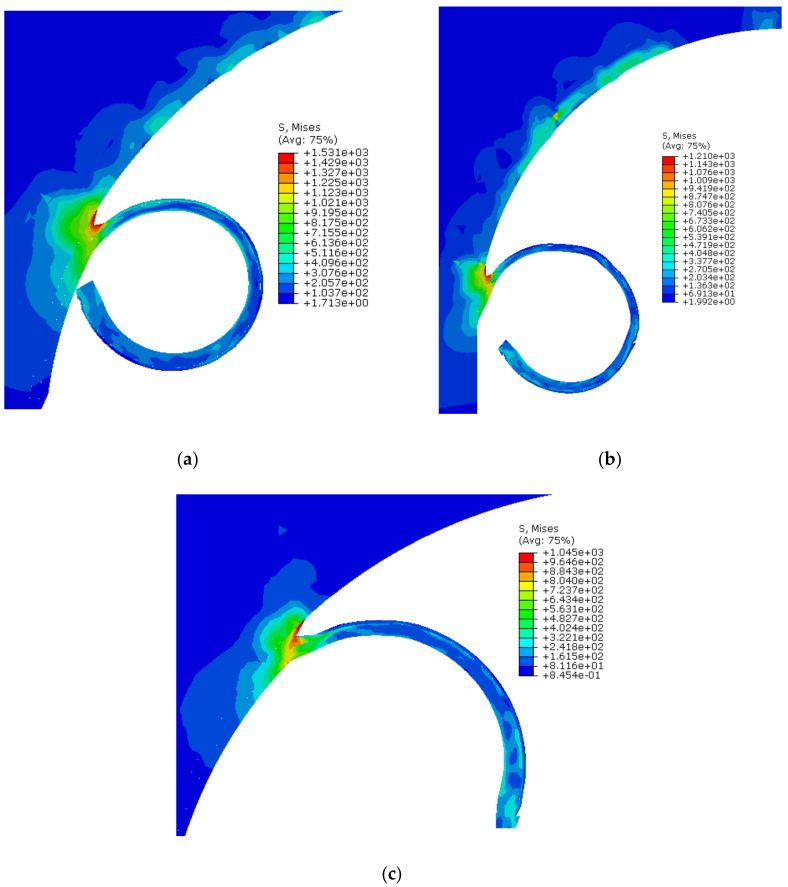
Chip flow stresses (Von Mises in MPa) at different cutting speeds. (**a**) Von Mises stress at 21 m/min; (**b**) Von Mises stress at 30 m/min; (**c**) Von Mises stress at 50 m/min.

For Ti6Al4V, the formation of the shear bands produced during the chip evolution process depends on the mesh density and damage constants used in the Johnson-Cook constitutive model. Moreover, cutting speed also plays an influential role in deciding chip morphology. In this study, stress on the deformed chip was analyzed against three different cutting speeds and contact friction coefficients. Under the specified cutting conditions, a continuous chip was observed. The chip produced during the numerical studies is shown in Figure 5. The peak stresses (Von Mises) observed during the cutting simulations are shown in Figure 5a (1531 MPa), b (1210 MPa) and c (1045 MPa) for 21, 30 and 50 m/min, respectively. This trend clearly indicates that the chip experiences more stress at low cutting speeds. The maximum stress in the deformed chip is observed at the contact shear zone. Similar findings were also reported by Asad et al., 2013 [43] and Saleem et al., 2017 [56].

The chips produced in this study are considerably smooth and do not show any prominent shear bands. The analyses show that the cutting tool takes more stress than the chip. This is because of the cutting tool geometry and mesh density. The research literature also highlighted this effect when a small change in the tool-tip nose, or the tool rake angle, produced considerably high stresses at the shear zone, hence causing substantial changes in chip morphology. From the deformed chip contours, it can be observed that the machining induced stress, instigated at the shear zone, propagates smoothly into the workpiece material up to a small thickness until a homogenized stress state is attained. This effect shows the compatibility of the material properties, damage parameters, and the contact conditions defined in the numerical model.

#### 3.1.3. Equivalent Plastic Strain

**Figure 6:**
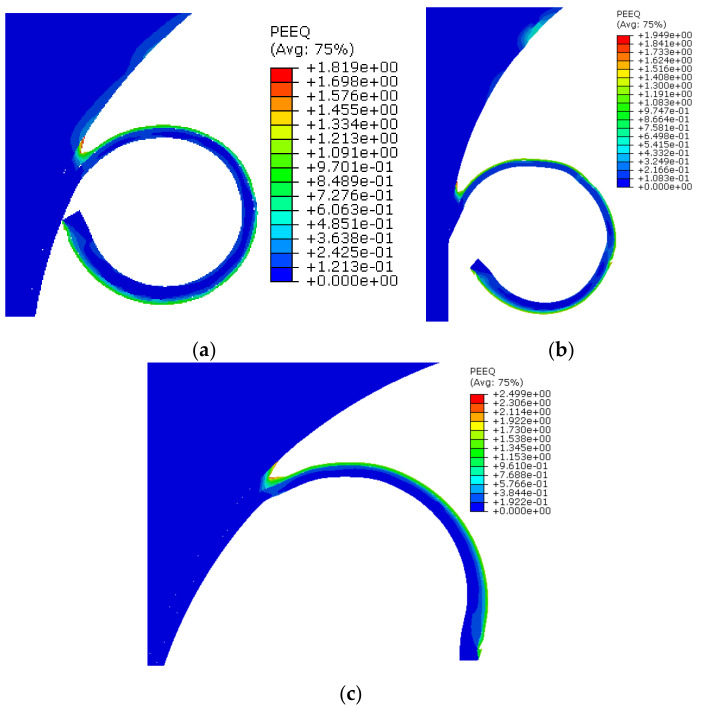
Equivalent plastic strains at the microchip zone. (**a**) Equivalent plastic strain at 21 m/min; (**b**) Equivalent plastic strain at 30 m/min; (**c**) Equivalent plastic strain at 50 m/min.

The contour plots of equivalent plastic strain at the microchip zone against three different cutting speeds are shown in Figure 6. The averaged plastic strains observed during the cutting speeds of 21, 30 and 50 m/min are 1.81, 1.94, and 2.49, respectively. The results indicate that plastic strain increases with the increase in cutting speed. However, for a deformed chip, the equivalent strain is influenced cumulatively by the cutting speed, as well as by the coefficient of contact friction. Therefore, in order to analyze the effect of both parameters, the values of the contact friction coefficients were taken as 0.2 for a cutting speed of 21 m/min, and 0.3 for cutting speeds of 30 and 50 m/min. The equivalent plastic strain of 1.81 (at 21 m/min) and 1.94 (at 30 m/min) were found to be consistent. However, the plastic strain increases rapidly to 2.49 with the cumulative effect of an increased in contact friction (0.3) and cutting speed (50 m/min). Published research studies have shown that the cutting edge geometry has a significant influence on the equipment plastic strain.

#### 3.1.4. Temperature at Chip-Tool Interface

**Figure 7:**
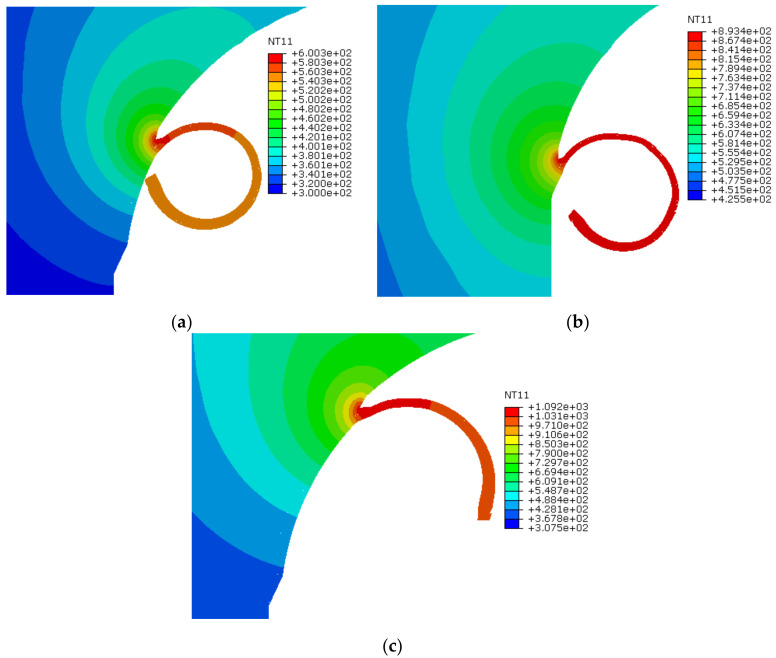
Temperatures at tool-chip interface in degree C. (**a**) NT at micro-chip zone at 21 m/min; (**b**) NT at micro-chip zone at 30 m/min; (**c**) NT at micro-chip zone at 50 m/min.

Figure 7 shows the temperatures encountered during the end milling of the Ti6Al4V alloy. The maximum temperatures (NT) observed at the tool-chip interface are shown in Figure 7a (600 °C), b (893 °C) and c (1092 °C) for 21, 30 and 50 m/min, respectively. With an increase in cutting temperature, the milling tool encounters more wear, which results in poor surface finish and subsequently enhanced heat-affected zones. This study investigated the influence of the contact friction coefficient, and cutting speed, on the heat generated during the cutting process. The cutting temperature rises substantially from 600 to 893 °C when both parameters (cutting speed and contact friction coefficient) increase simultaneously, that is, from 21 m/min (with 0.2) to 30 m/min (with 0.3). Due to the small-time step, it is difficult to estimate the exact time when the model attains the adiabatic conditions. It is also difficult to predict the precise temperature at the tool-chip interface at particular angular positions of the cutting flute.

## 4. Conclusions

From the presented research, pertinent conclusions can be drawn with respect to the machining parameters of the Ti6Al4V alloy:

1. The predicted cutting reaction force pattern demonstrates that the cutting reaction force stabilizes, and then reduces, with an increase in cutting speed. This is because of the steady cutting conditions. However, it is important to sustain a suitable depth of cut while increasing the speed. The predicted cutting forces correlate very well with the experimental data. The small deviations between simulated and experimental data are due to the limitations of the numerical model, which do not incorporate factors such as cutter vibrations, tool wear, and tool-spindle run-out effect. Furthermore, small inaccuracies are introduced due to the approximated trochoidal trajectory path.
2. This study also demonstrates that due to its low thermal conductivity, the Ti6Al4V alloy promotes heat concentration on the cutting tool with very high temperature increases in the tooltip area. This greatly impacts the surface integrity of the workpiece. In future research, the study of surface integrity and tribological properties will be implemented by undertaking the influence of different cooling and lubrication techniques [72,73]. The uniformity of predicted results at different cutting speeds validates the numerical cutting scheme, material properties applied, and constitutive relations selected for the Ti6Al4V alloy.
3. With an increase in cutting speeds, consistent trends were observed, allowing for accurate evaluations of the deformed chip, as well as the Von Mises stresses at the micro-chip zone, the resultant plastic strains, and the temperature distribution at the shearing zone.
4. The various outputs from the simulations can be combined in a manner that allows for estimates of the quality of the surface integrity for the workpiece. Consequently, this numerical model can contribute in defining the ultimate manufacturing production parameters for the machining of medical implants and devices.

## Figures and Tables

**Table 1:** Thermo-mechanical properties for Ti6Al4V [16,65].

Material Property	Workpiece		Cutting Tool (Tungsten Carbide)				
Density ρ (kg/m^3^)	4430		14500				
Elastic modulus E (GPa)	110		540				
Poisson ratio ν	0.33		0.227				
Thermal Conductivity (W/(m·K))	See below		84 (at 20°C), 63 (at 1000 °C)				
Specific Heat C_p_ (JKg^−1^ °C^−1^)	See below		220				
Material expansion coefficient α (µm/m^−1^ °C^−1^)	9		5.8				
T_melt_ (°C)	1630		-				
T_room_ (°C)	25		25				
Temperature-dependent properties (°C) [66]							
Temperature	20	500	995	1100	1200	1600	1700
Material Thermal conductivity λ (W/m^−1^ °C^−1^)	7	12.6	22.7	19.3	21	25.8	83.5
Material Specific heat C_p_ (JKg^−1^ °C^−1^)	546	651	753	641	660	732	831

**Table 2:** Johnson-Cook constants for Ti6Al4V [67].

A (MPa)	B (MPa)	n	C	m	D1	D2	D3	D4	D5
1098	1092	0.93	0.014	1.1	−0.09	0.25	−0.5	0.014	3.87

**Table 3:** Cutting simulation results.

Speed (V) m/min	μ	Cutting Force Avg (N)	Temperature (°C)	Chip Stress (MPa)	PEEQ Avg 75%
21	0.2	387	600.3	1531	1.81
30	0.3	401	893.4	1210	1.94
50	0.3	374	1092	1045	2.49

**Table 4:** Cutting reaction force with respect to time.

Time Step	Appx Cutter Position	Time	RF_V21_µ0.2	Time	RF_V30_µ0.3	Time	RF_V50_µ0.3
0	0	0.00000	0.005	0.00000	0.010	0.00000	0.027
1	4.5	0.00090	386.255	0.00070	465.004	0.00050	473.117
2	9	0.00180	369.599	0.00140	417.819	0.00100	422.312
3	13.5	0.00270	377.587	0.00210	379.486	0.00150	389.114
4	18	0.00360	405.505	0.00280	377.515	0.00200	397.512
5	22.5	0.00450	426.608	0.00350	376.450	0.00250	353.158
6	27	0.00540	383.301	0.00420	362.578	0.00300	329.536
7	31.5	0.00630	369.926	0.00490	472.197	0.00350	352.602
8	36	0.00720	384.778	0.00560	483.518	0.00400	361.417
9	40.5	0.00810	431.720	0.00630	393.827	0.00450	403.442
10	45	0.00900	302.955	0.00700	439.350	0.00500	374.224
11	49.5	0.00990	386.552	0.00770	485.474	0.00550	356.232
12	54	0.01080	368.700	0.00840	486.897	0.00600	374.998
13	58.5	0.01170	397.946	0.00910	327.058	0.00650	398.374
14	63	0.01260	421.719	0.00980	353.592	0.00700	424.472
15	67.5	0.01350	448.239	0.01050	439.376	0.00750	416.095
16	72	0.01440	472.954	0.01120	481.315	0.00800	446.659
17	76.5	0.01530	480.196	0.01190	437.673	0.00850	414.637
18	81	0.01620	405.807	0.01260	397.299	0.00900	417.390
19	85.5	0.01710	424.362	0.01330	453.786	0.00950	361.062
20	90	0.01800	482.547	0.01400	406.628	0.01000	390.120
Avg			387.012		401.755		374.119
